# Angiogenic Capacity of Dental Pulp Stem Cell Regulated by SDF-1*α*-CXCR4 Axis

**DOI:** 10.1155/2017/8085462

**Published:** 2017-05-15

**Authors:** Hyun Nam, Gee-Hye Kim, Yoon-Kyung Bae, Da-Eun Jeong, Kyeung-Min Joo, Kyunghoon Lee, Sun-Ho Lee

**Affiliations:** ^1^Department of Neurosurgery, Samsung Medical Center, Sungkyunkwan University School of Medicine, Seoul 06351, Republic of Korea; ^2^Stem Cell and Regenerative Medicine Center, Research Institute for Future Medicine, Samsung Medical Center, Seoul 06351, Republic of Korea; ^3^Single Cell Network Research Center, Sungkyunkwan University School of Medicine, Suwon 16419, Republic of Korea; ^4^Laboratory of Molecular Genetics, Dental Research Institute, School of Dentistry, Seoul National University, Seoul 03080, Republic of Korea; ^5^Department of Health Sciences and Technology, SAIHST, Sungkyunkwan University, Seoul 06351, Republic of Korea; ^6^Department of Anatomy & Cell Biology, Sungkyunkwan University School of Medicine, Suwon 16419, Republic of Korea; ^7^Center for Molecular Medicine, Samsung Biomedical Research Institute, Sungkyunkwan University School of Medicine, Suwon 16419, Republic of Korea

## Abstract

Previously, the perivascular characteristics of dental pulp stem cells (DPSCs) were reported, which suggested the potential application of DPSCs as perivascular cell source. In this study, we investigated whether DPSCs had angiogenic capacity by coinjection with human umbilical vein endothelial cells (HUVECs) in vivo; in addition, we determined the role of stromal cell-derived factor 1-*α* (SDF-1*α*) and C-X-C chemokine receptor type 4 (CXCR4) axis in the mutual interaction between DPSCs and HUVECs. Primarily isolated DPSCs showed mesenchymal stem cell- (MSC-) like characteristics. Moreover, DPSCs expressed perivascular markers such as NG2, *α*-smooth muscle actin (*α*-SMA), platelet-derived growth factor receptor *β* (PDGFR*β*), and CD146. In vivo angiogenic capacity of DPSCs was demonstrated by in vivo Matrigel plug assay. We could observe microvessel-like structures in the coinjection of DPSCs and HUVECs at 7 days postinjection. To block SDF-1*α* and CXCR4 axis between DPSCs and HUVECs, AMD3100, a CXCR4 antagonist, was added into Matrigel plug. No significant microvessel-like structures were observed at 7 days postinjection. In conclusion, DPSCs have perivascular characteristics that contribute to in vivo angiogenesis. The findings of this study have potential applications in neovascularization of engineered tissues and vascular diseases.

## 1. Introduction

Dental pulp stem cells (DPSCs) are stem cells residing in the dental pulp [1]. The functional role of DPSCs is the regeneration of damaged dental pulp based on their potential to make dentin-pulp-like structures in vivo [1]. DPSCs have mesenchymal stem cell- (MSC-) like characteristics including the expression of surface antigens and in vitro differentiation potentials [1]. Recently, stem cells derived from teeth also have pericyte-like characteristics [2–4], which is in accordance with the previous reports suggesting that the origin of MSCs is the perivascular region [5, 6].

Angiogenesis is defined as the formation of new vessels from preexisting blood vessels and is mediated by the mutual interactions between pericytes, also called perivascular cells and endothelial cells [7, 8]. Angiogenesis has important roles in tissue regeneration and repair. Especially, tissue engineering requires rapid formation of vascular networks with the host circulatory system, in order to satisfy necessary supply of oxygen and nutrients, and removal of waste products [9]. To overcome the problem of vessel formation, several strategies have been suggested including delivery of angiogenic molecules. However, there are limitations involved in the vascularization of thick engineered tissues [7, 8]. Recently, it was suggested that coimplantation of perivascular cells and endothelial progenitor cells (EPCs) could form functional microvessels in vivo, which might be useful for tissue engineering [10, 11].

The stromal cell-derived factor 1 (SDF-1), also called as CXCL12, is one of CXC chemokines that transduces signaling through binding to CXCR4 [12, 13]. SDF-1*α*-CXCR4 axis is known to regulate hematopoiesis [14, 15] and is involved in the homing and engraftment of transplanted hematopoietic stem/progenitor cells (HSPCs) in recipient bone marrow [16]. Moreover, SDF-1*α*-CXCR4 axis has been focused on the migration of stem cells toward injured tissues or organs [17, 18]. DPSCs are also affected by SDF-1*α*-CXCR4 axis, which is involved in the proliferation, differentiation, and recruitment of DPSCs [19–21].

DPSCs are reported to have perivascular characteristics; hence, DPSCs could be a feasible source of perivascular cells contributing to in vivo angiogenesis with endothelial cells. Recently, the angiogenic potential of DPSCs via secretory angiogenic factors has been established [3, 22–24]. However, whether DPSCs are directly involved in in vivo angiogenesis as perivascular cells and the mutual interactions between DPSCs and human umbilical vein endothelial cells (HUVECs) during in vivo angiogenesis remains unclear. In this study, we investigated the perivascular characteristics of DPSCs and their functional involvement in in vivo angiogenesis with HUVECs. Moreover, we determined the role of the SDF-1*α*-CXCR4 axis in mutual interactions between DPSCs and HUVECs.

## 2. Materials and Methods

### 2.1. Primary Isolation and Culture

This study was reviewed and approved by the Institutional Review Board of Samsung Medical Center (IRB file number 2016-09-120). Human third molars were delivered in sterile saline. Primary isolation and culture of DPSCs were conducted as described in a previous report [25]. Dental pulp tissues were extracted and mechanically dissociated in the enzyme solution containing 1 mg/mL of collagenase type I (Gibco, Grand Island, NY, USA) and 2.4 mg/mL of dispase (Gibco) at 37°C for 1 hour. After enzyme inactivation with *α*-MEM (HyClone, Road Logan, Utah, USA) supplemented with 10% FBS (HyClone), the cells were washed twice with *α*-MEM (HyClone, Road Logan, Utah, USA). Single-cell suspensions were maintained in *α*-MEM supplemented with 10% FBS (HyClone). The medium was replaced every 3 days. The cells were subcultured at 70% confluency. Before passage, cells were photographed and counted to calculate population doubling length. HUVECs were commercially purchased (Lonza, Walkersville, MD, USA) and cultured in EGM-2 (Lonza) until the 6th passage. All experiments were conducted using DPSCs at passage 3 and HUVECs at passage 6.

### 2.2. FACS Analysis

The cells were detached and resuspended in Dulbecco's phosphate-buffered saline (DPBS; Welgene, Dae-gu, Korea) and 2% FBS. About 1.0 × 10^5^ cells were applied with antibodies for 30 minutes on ice. The antibodies are listed in Supplementary Table 1 available online at https://doi.org/10.1155/2017/8085462. After washing, the fluorescence intensity was determined by a FACS Calibur (Becton Dickinson, San Jose, CA, USA). For the analysis of data, we used FLOWJO software (Tree Star Inc., Ashland, OR, USA).

### 2.3. In Vitro Differentiation of DPSCs

Cells were cultured to be confluent for osteogenic and adipogenic differentiation. Osteogenic medium consisted of *α*-MEM and 5% FBS supplemented with 50 *μ*g/mL L-ascorbic acid phosphate, 10 mM *β*-glycerophosphate, and 0.1 *μ*M dexamethasone (all from Sigma-Aldrich, St. Louis, MO, USA). Adipogenic medium included *α*-MEM and 5% FBS supplemented with 10 *μ*g/mL insulin, 50 *μ*M indomethacin, 0.5 mM isobutylmethaylxanthine, and 1 *μ*M dexamethasone (all from Sigma-Aldrich). Cells were cultured for 21 days in differentiation medium, and fresh medium was replaced every other days. Calcium deposit and lipid vacuoles were stained with Alizarin red solution and Oil red O solution (all from Sigma-Aldrich), respectively. To induce chondrogenic differentiation, 1 × 10^4^ cells were centrifuged at 500*g* for 5 minutes at 4°C in a 15 mL conical tube. Chondrogenic medium consisted of high-glucose DMEM supplemented with 50 *μ*g/mL ascorbate-2-phosphate, 100 *μ*g/mL sodium pyruvate, 40 *μ*g/mL L-proline, 1% ITS + Premix (all Sigma-Aldrich), and 10 ng/mL TGF-*β*3 (PeproTech, Rocky Hill, USA). Cells were cultured for 21 days, and fresh medium was replaced every other day. Spheroid-like structure was fixed with 4% paraformaldehyde (PFA) (Sigma-Aldrich) and embedded in paraffin. Slides were prepared from 5 *μ*m thick sections. Alcian blue staining (Sigma-Aldrich) was used to assess chondrogenic differentiation.

### 2.4. Quantitative PCR (qPCR)

The total RNA was extracted from DPSCs and HUVECs using an RNeasy Mini Kit (Qiagen, Valencia, CA, USA). cDNA was generated by reverse transcription of total RNA (2 *μ*g) using SuperScript III (Invitrogen TM, Carlsbad, CA, USA) following the manufacturer's protocol. Reaction mixture (20 *μ*L) for qPCR included cDNA for each primer (Supplementary Table 2) and THUNDERBIRD SYBR qPCR Mix (QPS-201, TOYOBO, Japan). qPCR was performed using Applied Biosystems 7500 Real-Time PCR System (Applied Biosystems, Foster City, CA, USA).

### 2.5. Semiquantitative PCR

PCR was performed with i-MAXII (Intron, Sungnam, Korea). The primer sequences of each primer are listed in Supplementary Table 2. Reaction condition included initial denaturation at 95°C for 2 minutes, followed by 35 cycles of denaturation at 95°C for 20 seconds, annealing at 60°C for 15 seconds, and extension at 72°C for 30 seconds. The number of PCR cycles for *GAPDH* was 25. The PCR products (2 *μ*L of total 20 *μ*L) were separated in 2% agarose gel with ethidium bromide.

### 2.6. In Vivo Matrigel Plug Assay

All animal study was conducted according to the Institutional Animal Care and Use Committee (IACUC) of Samsung Biomedical Research Institute (SBRI, Seoul, Korea) (20161019001). We conducted in vivo Matrigel plug assay based on previous reports [4, 10, 26]. Adult male balb-c/nu mice (6–8-week old) were purchased (Orient Bio., Seongnam, Korea). Total 2.0 × 10^6^ cells were prepared for one injection via resuspending cells in 200 *μ*L of ice-cold Matrigel (BD Bioscience, San Jose, CA, USA). The ratios of DPSCs and HUVECs were 100 : 0, 50 : 50, and 0 : 100 (DPSCs : HUVECs). The mixture was injected subcutaneously into the dorsal surface of mice using a 25-gauge needle. Mice injected with Matrigel alone served as controls. One implant was injected per mouse, and three mice were injected for each group. To inhibit SDF-1*α* and CXCR4 axis, 10 *μ*M of AMD3100 (Sigma-Aldrich) was added into Matrigel plug before injection. At 7 days postinjection, mice were euthanized using CO_2_. Matrigel plug was removed and fixed in 10% buffered formalin overnight at room temperature.

### 2.7. Histology

Formalin-fixed Matrigel plug was embedded in paraffin. Slides were prepared from 5 *μ*m thick sections. For histological analysis, hematoxylin (Sigma-Aldrich) and eosin (Sigma-Aldrich) staining was applied.

### 2.8. Immunofluorescent Staining

The sections were deparaffinized and rehydrated. Endogenous peroxidase was inactivated with 10% hydrogen peroxide for 10 minutes. The antigens were retrieved by pepsin for 10 minutes at 37°C. The sections were blocked for 30 minutes in 10% normal goat serum (Jackson ImmunoResearch Inc., West Grove, PA, USA), followed by incubation with mouse anti-*α* smooth muscle actin (1 : 500; Sigma-Aldrich) and rabbit anti-human CD31 (1 : 50; Santa Cruz Biotechnology) for 1 hour at room temperature. After washing, the slides were treated with secondary antibodies including Alexa 488-conjugated goat anti-mouse IgG (1 : 1000; Invitrogen) and Alexa 594-conjugated goat anti-rabbit IgG (1 : 1000; Invitrogen) for 1 hour at room temperature. Nuclei were stained with DAPI (Sigma-Aldrich). The slides were observed using a confocal laser scanning microscope (Fluoview FV 300, Olympus, Japan).

### 2.9. Preparation of Conditioned Medium from DPSC (DPSC-CM)

DPSCs (3 × 10^5^ cells per dish) were seeded in 100 mm dish and cultured to be 80% confluency. After washing with PBS two times, cells were cultured in endothelial basal medium (EBM) (Lonza) for 24 hours. Conditioned medium was collected into a 50 mL conical tube and centrifuged at 900*g* for 10 minutes at 4°C. Supernatant was transferred into a new 50 mL conical tube and used for further experiments.

### 2.10. Wound Healing Assay

HUVECs (5 × 10^5^ per well) were seeded in 12-well plates and grown overnight. After cells had completely adhered to the plates, one vertical line was scraped in each well using a yellow pipette tip. After scraping, cells were washed with PBS and then incubated with various concentrations of DPSC-CM. To inhibit SDF-1*α* and CXCR4 axis, 10 *μ*M of AMD3100 (Sigma-Aldrich) was added into the medium. Images were obtained at different time intervals using a microscope video system. The widths of each line at the three different points were measured and averaged. Migratory ability was calculated using the following formula: {(width at 0 hours − width at 9 hours)/width at 0 hours} × 100%.

### 2.11. Apoptosis Assay

HUVECs (3 × 10^3^ cells per well) were seeded in 96-well plates. After 24 hours of incubation, cells were incubated with various concentrations of DPSC-CM without FBS. To inhibit SDF-1*α* and CXCR4 axis, 10 *μ*M of AMD3100 (Sigma-Aldrich) was added into medium. EZ-CYTOX solution (10 *μ*L for each well) (Daeil Lab Service Co., Seoul, Korea) was added into each well and incubated for 2 hours. The absorbance of each well was determined at 450 nm using an ELISA reader (Bio Tek Instruments, Burlington, VT, USA).

### 2.12. Statistics

Experimental data were analyzed using Student's *t*-test (two-tailed). When *p* values were less than 0.05, the data was considered as statistically significant.

## 3. Results

### 3.1. Primary Isolation and Characterization of DPSCs

**Figure 1:**
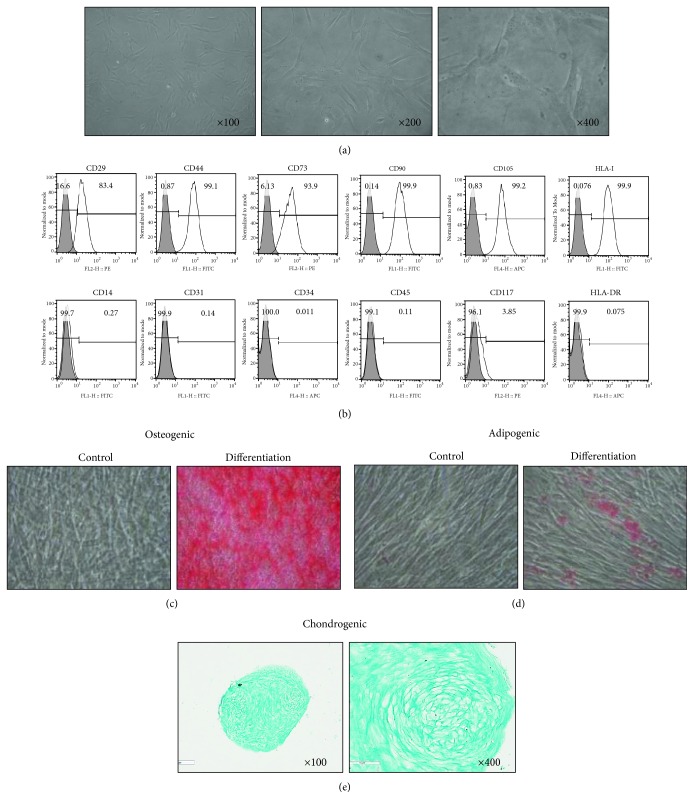
Primary isolation and characterization of DPSCs. Primary isolated DPSCs were cultured and characterized. (a) DPSCs showed typical MSC-like morphology at passage 3. (b) The expression of surface antigens was determined by FACS analysis. DPSCs were positive for MSC markers (CD29, CD44, CD73, CD90, and CD105) but negative for hematopoietic cell markers (CD14, CD34, CD45, CD117, and HLA-DR) and endothelial cell marker (CD31). DPSCs were induced in osteogenic or adipogenic medium for 21 days. (c) Alizarin red staining revealed deposits of calcium. (d) Oil red O staining showed lipid vacuoles. (e) Alcian blue staining confirmed chondrogenic differentiation.

Primary isolated DPSCs showed MSC-like characteristics. Although the morphology of DPSCs was heterogeneous, most DPSCs showed MSC-like bipolar morphology (Figure 1(a)). The growth rate of DPSCs was linear, without growth regression during the culture period (data not shown). The expression pattern of surface antigen was analyzed by FACS analysis. DPSCs were positive for CD29, CD44, CD73, CD90, and CD105 but negative for CD14, CD31, CD34, CD45, CD117, and HLA-DR (Figure 1(b)). To determine in vitro differentiation potentials, DPSCs were cultured to confluence, and culture medium was changed to osteogenic, adipogenic, or chondrogenic medium for 21 days. Alizarin red and Oil red O staining revealed deposits of calcium and lipid vacuoles, respectively (Figures 1(c) and 1(d)). Alcian blue staining confirmed chondrogenic differentiation (Figure 1(e)).

### 3.2. The Perivascular Characteristics of DPSCs

**Figure 2:**
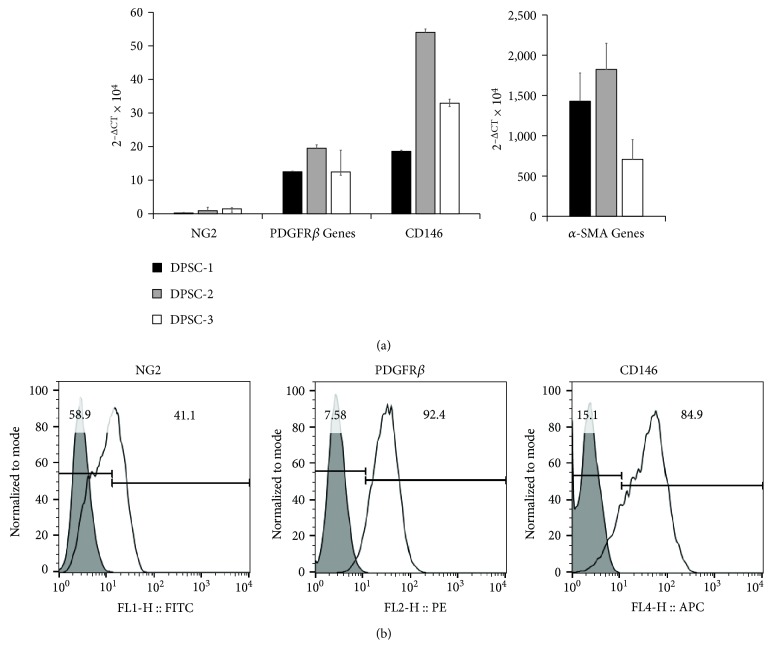
The expression of perivascular markers in DPSCs. The expression of perivascular markers in three different lines of DPSCs was determined by qPCR and FACS analysis. (a) DPSCs expressed *NG2*, *α-SMA*, *PDGFRβ*, and *CD146*. Arbitrary unit in *y*-axis represented 2^−∆CT^ × 10^4^. (b) In the results by FACS analysis, DPSCs expressed NG2, PDGFR*β*, and CD146. However, the expression of NG2 was subdivided into NG2-positive and NG2-negative populations dependent on the lines of DPSCs. The expression of CD146 was broadly distributed. One of representative pieces of data was shown.

To determine perivascular characteristics of three different lines of DPSCs, the expression of perivascular markers was determined. In the results of qPCR, all three lines of DPSCs expressed *α*-smooth muscle actin (*α-SMA*), platelet-derived growth factor receptor *β* (*PDGFRβ*), and *CD146* at different levels (Figure 2(a)). The expression level of *α*-SMA was highest among them (Figure 2(a) and Supplementary Figure 1). The expression of perivascular markers was confirmed by FACS analysis (Figure 2(b)). DPSCs expressed NG2, PDGFR*β*, and CD146. The expression of NG2 was subdivided into positive and negative populations, and that of CD146 was broadly distributed.

### 3.3. In Vivo Angiogenesis

**Figure 3:**
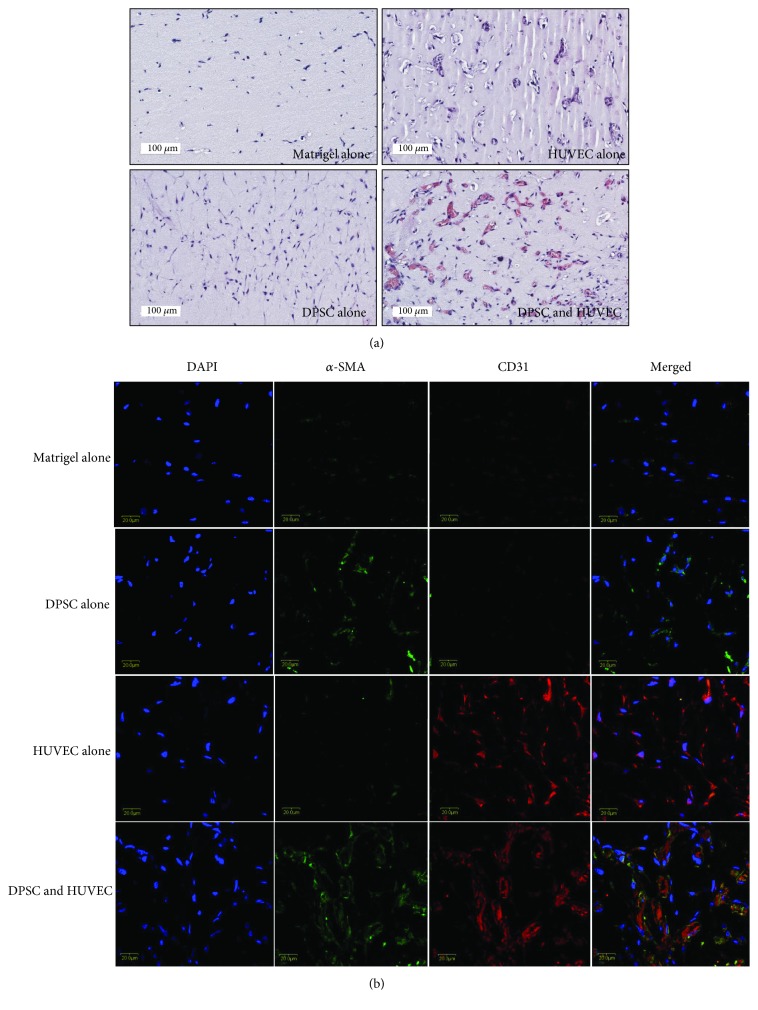
In vivo angiogenic potential of DPSCs. To investigate in vivo angiogenic potential of DPSCs, Matrigel plug assay was conducted. DPSCs and HUVECs were subcutaneously injected and separated or together into immunodeficient mice. At 7 days postinjection, Matrigel plug was removed and analyzed by H&E and immunofluorescent stainings. (a) In the results of DPSCs alone or HUVEC alone, no obvious microvessel-like structures were observed. However, when DPSCs and HUVECs were coinjected, microvessel-like structures were formed and red blood cells were observed in the lumen. (b) Immunofluorescent staining by CD31 and *α*-SMA showed that microvessel-like structures were stained on coinjection with DPSCs and HUVECs subcutaneously.

We further verified the functional involvement of DPSCs as perivascular cells in in vivo angiogenesis. To determine the angiogenic capacity of DPSCs, in vivo Matrigel plug assay was performed. Matrigel mixture containing DPSCs alone, HUVECs alone, or the combination of DPSCs and HUVECs was prepared and injected into the dorsal region of immunodeficient mice. At 7 days postinjection, Matrigel plug was analyzed by hematoxylin and eosin (H&E) staining. Histological analysis showed that the injection of Matrigel alone did not contain typical microvessel-like structures (Figure 3(a)). Moreover, Matrigel with DPSCs alone or HUVECs alone showed no significant microvessel-like structures (Figure 3(a)). However, when DPSCs were injected with HUVECs, a robust generation of microvessel-like structures was observed. Murine red blood cells and white blood cells within the microvessel-like structures suggested that the newly formed microvessels anastomosed with host vasculature and were perfused. Next, we analyzed the localization of injected DPSCs and HUVECs. We observed that *α*-SMA-positive DPSCs and CD31-positive HUVECs were localized around microvessel-like structures (Figure 3(b)).

### 3.4. SDF-1*α* and CXCR4 Axis during In Vivo Angiogenesis

**Figure 4:**
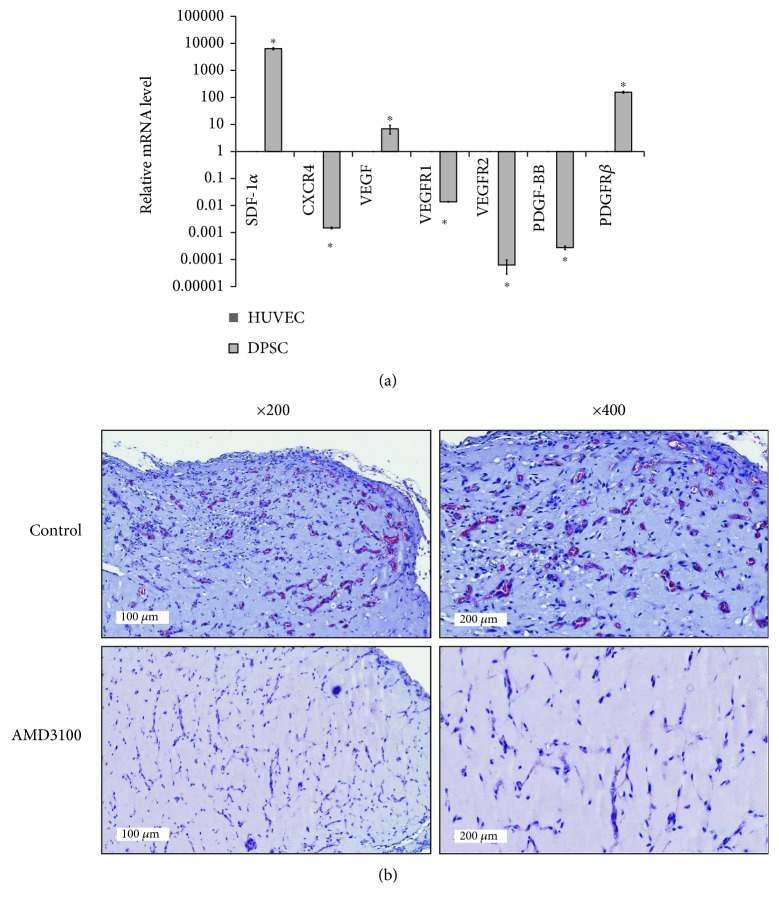
The involvement of SDF-1*α* and CXCR4 axis in in vivo angiogenesis by DPSCs and HUVECs. The expression of angiogenic factors and receptors was verified by qPCR. (a) The expression of *SDF-1α*, *PDGFRβ*, and *VEGF* was higher in DPSCs than that in HUVECs. On the contrary, the expression of *CXCR4*, *PDGF-BB*, *VEGFR1*, and *VEGFR2* was higher in HUVECs than that in DPSCs. ^∗^*p* < 0.05. (b) To confirm the functional involvement of SDF-1*α* and CXCR4 axis in in vivo angiogenesis, AMD3100, an antagonist of CXCR4, was mixed with Matrigel plug. At 7 days postinjection, there were no microvessel-like structures in the AMD3100-treated group as compared to control group.

To investigate mutual interactions between DPSCs and HUVECs, the expression levels of representative angiogenic factors and their receptors were determined by qPCR. The mRNA expression of *SDF-1α*, *PDGFRβ*, and *VEGF* in DPSCs was higher than that in HUVECs (Figure 4(a)). On the other hand, the mRNA expression of *CXCR4*, *PDGF-BB VEGFR1*, and *VEGFR2* was higher in HUVECs than that in DPSCs (Figure 4(a)). These contradictory expression patterns were suggestive of mutual interactions between DPSCs and HUVECs. Among them, the functional involvement of SDF-1*α*-CXCR4 axis was investigated by using AMD3100, an antagonist of CXCR4. AMD3100 was mixed with DPSCs and HUVECs in Matrigel plug assay. At 7 days postinjection, H&E staining of the coinjection of DPSCs and HUVECs indicated an adequate number of microvessel-like structures. However, no microvessel-like structures were observed in the AMD3100-treated group (Figure 4(b)). To verify the in vivo angiogenic effects of DPSCs, we investigated the effects of DPSC-CM on the migration and survival of HUVECs in vitro. We could confirm that DPSC-CM could increase the migration and survival of HUVECs (Supplementary Figures 2 and 3). Moreover, these beneficial effects of DPSC-CM were reduced by AMD3100.

## 4. Discussion

Teeth contain various types of stem cells. DPSCs, one of the well-established stem cells derived from teeth, are considered to have similar characteristics to MSCs [1]. The perivascular region has recently been identified as the origin of MSCs [5]. A previous report suggested that DPSCs may also be localized in the perivascular region and express perivascular markers [27]. Pericytes, also called perivascular cells, are located within the basement membrane of vessels and interact with endothelial cells to regulate the physiology of blood vessels [28, 29]. Although the specific markers for perivascular cells have not been identified, combined expression of perivascular markers in DPSCs suggest that they might have perivascular characteristics and originate from the perivascular region. In accordance to previous reports, we showed that DPSCs expressed perivascular markers including *α*-SMA, NG2, PDGFR*β*, and CD146. According to our results, primary isolated DPSCs expressed different levels of perivascular markers, suggestive of their perivascular origin and heterogeneous population. Unexpectedly, there was different expression level of perivascular markers between qPCR data and FACS data. This could be explained by different posttranscriptional regulation between mRNA and protein [30].

The main recovery mechanisms of MSCs are mediated by secreted factors, which are considered as paracrine effects [31, 32]. After the transplantation of MSCs into damaged tissues or organs, they could not be maintained for a life-long time but beneficial effects were observed. Angiogenesis is one of the important recovery mechanisms of damaged organs, and MSCs have secretion of various types of angiogenic cytokines and chemokines [33]. Recently, the angiogenic potential of DPSCs was confirmed by indirect methods including in vitro tube formation, chicken chorioallantoic membrane (CAM) assay, or in vivo tooth slice-based model [3, 22–24, 34, 35]. These angiogenic mechanisms can be explained by the beneficial angiogenic factors secreted by DPSCs. However, for better understanding the mutual interactions between perivascular cells and endothelial cells, an appropriate system is necessary such as in vivo Matrigel plus assay. Two types of cells are required for in vivo Matrigel plug assay including perivascular cells and endothelial (progenitor) cells for optimal microvessel-like structure formation within Matrigel [10, 11]. Therefore, in vivo Matrigel plug assay may be useful to confirm in vivo angiogenic potential of DPSCs as a perivascular cell source. In the injection groups of DPSCs alone or HUVECs alone, there were no significant microvessel-like structures in vivo. This result is in accordance with a previous report, which suggested the necessity of the coinjection of perivascular cells and endothelial cells [10, 26]. When DPSCs and HUVECs were coinjected into immunodeficient mice, microvessel-like structures were readily observed with host blood cells in the lumen. Moreover, immunofluorescent staining by CD31 and *α*-SMA showed that DPSCs were colocalized within a perivascular region near HUVECs. We could not exclude the possibility of the recruitment of host-derived *α*-SMA- or CD31-positive cells within microvessel-like structures. However, these data suggested that DPSCs could have a functional role as perivascular cells for in vivo angiogenesis. Further studies are required to identify the mutual interactions between DPSCs and HUVECs.

SDF-1*α* and CXCR4 axis is important to signaling pathways in neovascularization including embryonic vasculogenesis and cancer [36, 37]. Recently, the beneficial roles of SDF-1*α* were reported in the neovascularization in cardiac infarct [38] and regeneration process in spinal cord injury [39], which indicates that increasing SDF-1 levels at the injury site enhances stem cell recruitment. In our study, DPSCs showed high expression of SDF-1*α* and low expression of CXCR4, corroborating a previous report [19, 21, 40]. These reports collectively suggest the important roles of SDF-1*α* expressed from DPSCs. On the other hand, HUVECs showed high expression of CXCR4 but low expression of SDF-1*α*. This suggested the functional roles of SDF-1*α* and CXCR4 axis between DPSCs and HUVECs. Treatment with AMD3100, a CXCR4 antagonist, reduced in vivo microvessel formation in Matrigel plug assay, thus confirming the involvement of SDF-1*α* and CXCR4 axis in in vivo microvessel-like structure formation by DPSCs and HUVECs. Moreover, we confirmed the beneficial effects of DPSC-CMs on the migration and survival of HUVECs by DPSC-CM, suggestive of angiogenic capacity of DPSCs. These data suggested that SDF-1*α*-CXCR4 axis might be involved in in vivo angiogenesis by DPSCs and HUVECs.

DPSCs are multipotent stem cells with beneficial paracrine effects such as angiogenesis and immune modulation [22, 41]. Moreover, the proliferation potential of DPSCs is comparable to other types of MSC-like cells [1]. Despite the availability and the stemness of DPSCs, the potential applications and preclinical efficacy of DPSCs have been limited to the regeneration of pulp and dentin [42]. In this study, we further investigated the applicability of DPSCs in in vivo angiogenesis, which is one of the main huddles to overcome for successful tissue-engineered constructs. Our study revealed that DPSCs could be a potential perivascular sources to form functional microvessel-like structures in vivo. The SDF-1*α* and CXCR4 axis seems to be an important mediator of in vivo angiogenesis, although other types of angiogenic factors from DPSCs could be helpful for in vivo microvessel-like organization with HUVECs. Considering the importance of vessel formation in tissue engineering, DPSCs are potentially important candidates for tissue engineering requiring enough blood supply as well as disordered vessel diseases such as cerebrovascular diseases.

## Supplementary Material

Supplementary Figure 1. The expression pattern of perivascular markers in DPSCs. After completion of semi-quantitative PCR, PCR products for each gene were separated and visualized. Supplementary Figure 2. The effect of DPSC-CM on the migration of HUVECs. The migration of HUVECs by DPSC-CM was determined by wound healing assay. After culturing of HUVECs with various concentrations of DPSC-CM (0%, 25%, 50%, and 100%) for 9 hours, the migration ability of HUVECs was analyzed. (A) The migration of HUVECs was increased in the dose-dependent manner of DPSC-CM.(B) When 10 μM AMD3100 was treated with DPSC-CM, we could observe decreased migration ability of HUVECs.(C) Relative wound closure was calculated. ∗P<0.05, significantly different from 10 μM AMD3100 treatment. Supplementary Figure 3. The effect of DPSC-CM on the survival of HUVECs. The survival of HUVECs by DPSC-CM was determined by apoptosis assay.(A) After culturing of HUVECs with various concentrations of DPSC-CM (0%, 25%, 50%, and 100%), the survival ability of HUVECs was analyzed. DPSC-CM could increase survival of HUVECs in dose-dependent manner. Y-axis was arbitrary unit which was normalized with the value at day 0. #P<0.05, significantly different from 0% DPSC-CM; ∗P<0.05, significantly different from 100% DPSC-CM(B) The effect of each concentration of DPSC-CM on the survival of HUVECs was shown separately. ∗P<0.05, significantly different from 10 μM AMD3100 treatment.Supplementary Table 1. Antibodies for FACS analysis. Supplementary Table 2. Primers for qPCR.
