# Supplementary material for: Angiogenic Capacity of Dental Pulp Stem Cell Regulated by SDF-1α-CXCR4 Axis
Source: Stem Cells Int. 2017 May 15;2017:8085462. doi: 10.1155/2017/8085462 (PMC5447288; doi:10.1155/2017/8085462)
Supplement: Supplementary file 1 — Supplementary Figure 1. The expression pattern of perivascular markers in DPSCs. After completion of semi-quantitative PCR, PCR products for each gene were separated and visualized. Supplementary Figure 2. The effect of DPSC-CM on the migration of HUVECs. The migration of HUVECs by DPSC-CM was determined by wound healing assay. After culturing of HUVECs with various concentrations of DPSC-CM (0%, 25%, 50%, and 100%) for 9 hours, the migration ability of HUVECs was analyzed. (A) The migration of HUVECs was increased in the dose-dependent manner of DPSC-CM.(B) When 10 μM AMD3100 was treated with DPSC-CM, we could observe decreased migration ability of HUVECs.(C) Relative wound closure was calculated. ∗P<0.05, significantly different from 10 μM AMD3100 treatment. Supplementary Figure 3. The effect of DPSC-CM on the survival of HUVECs. The survival of HUVECs by DPSC-CM was determined by apoptosis assay.(A) After culturing of HUVECs with various concentrations of DPSC-CM (0%, 25%, 50%, and 100%), the survival ability of HUVECs was analyzed. DPSC-CM could increase survival of HUVECs in dose-dependent manner. Y-axis was arbitrary unit which was normalized with the value at day 0. #P<0.05, significantly different from 0% DPSC-CM; ∗P<0.05, significantly different from 100% DPSC-CM(B) The effect of each concentration of DPSC-CM on the survival of HUVECs was shown separately. ∗P<0.05, significantly different from 10 μM AMD3100 treatment.Supplementary Table 1. Antibodies for FACS analysis. Supplementary Table 2. Primers for qPCR. [file 8085462.f1.pdf]

Supplementary Figure 1.

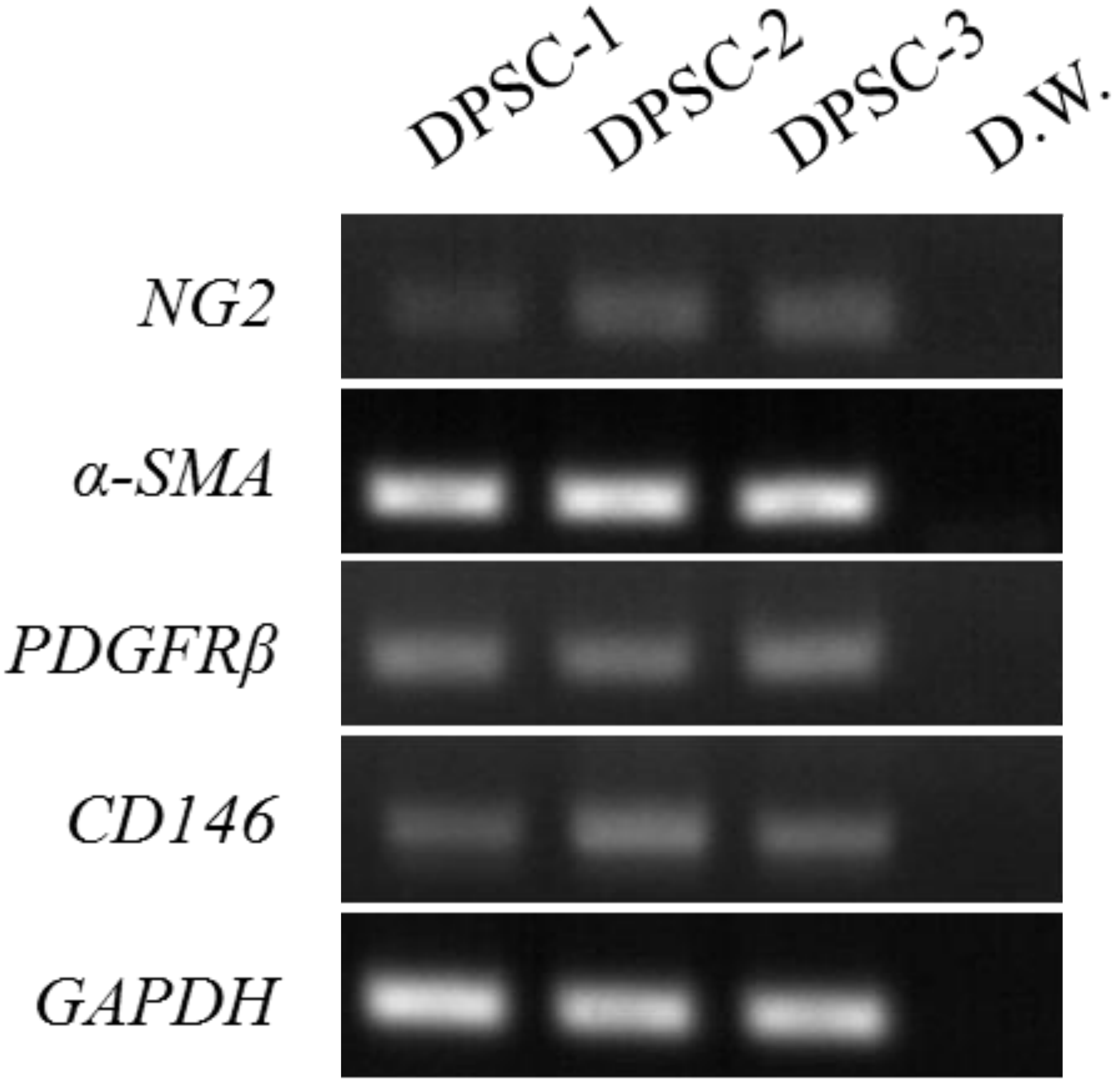

Supplementary Figure 2.

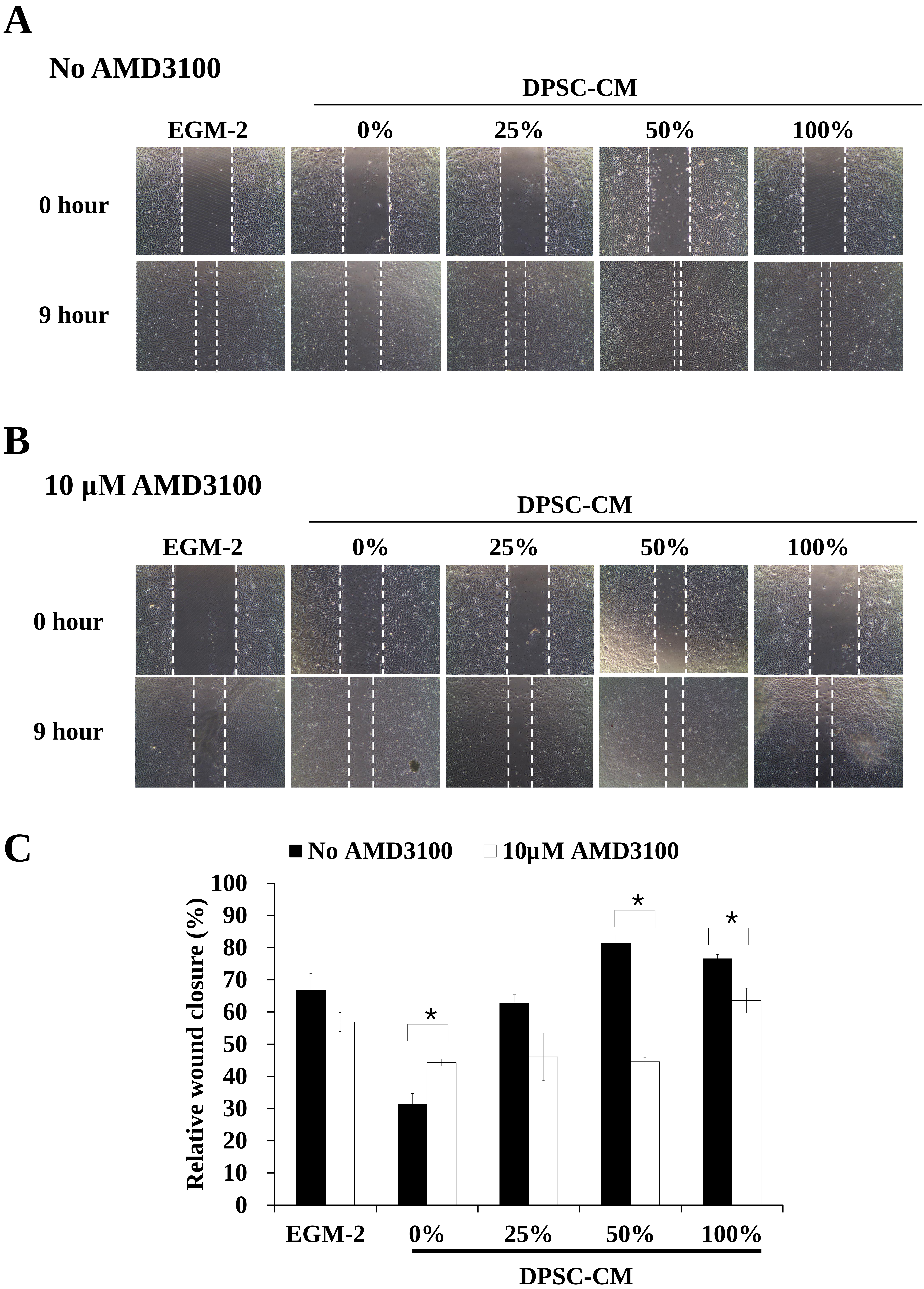

Supplementary Figure 3.

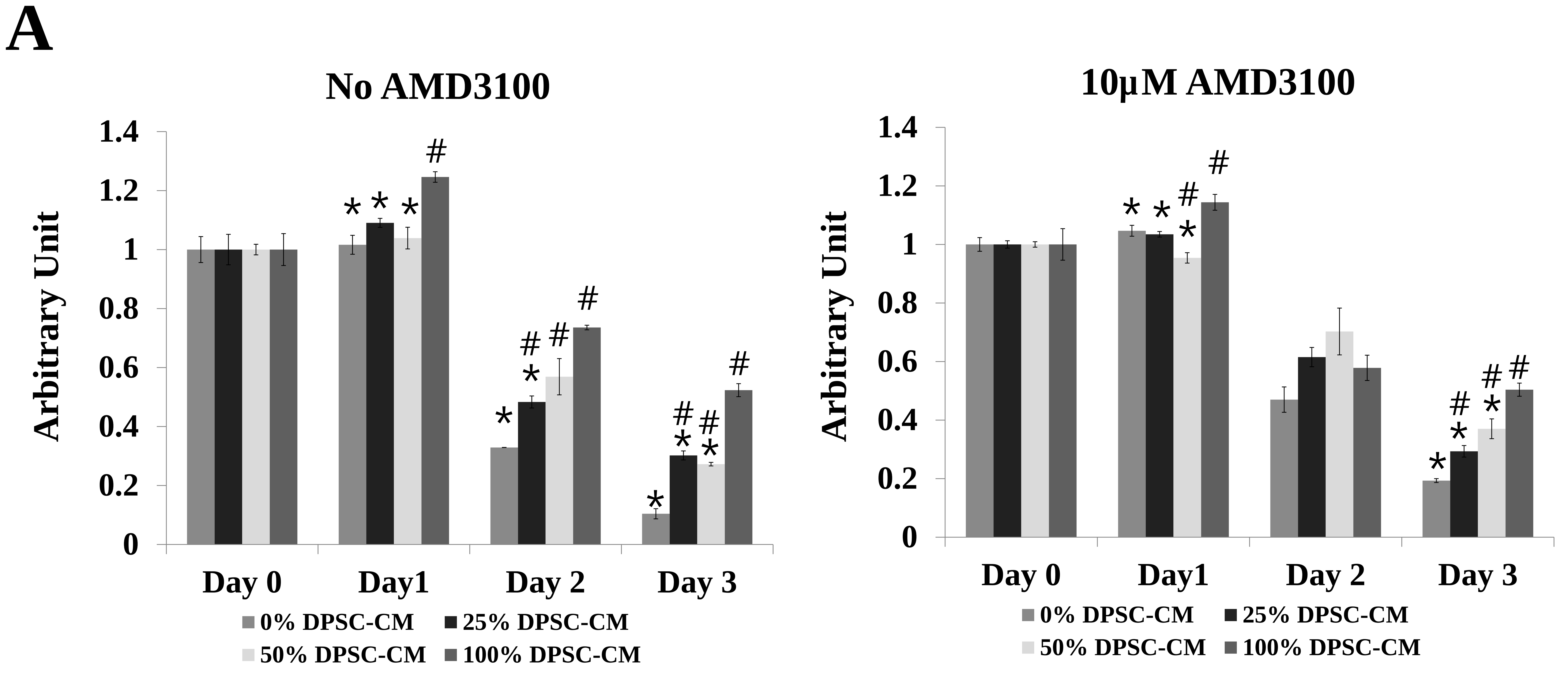

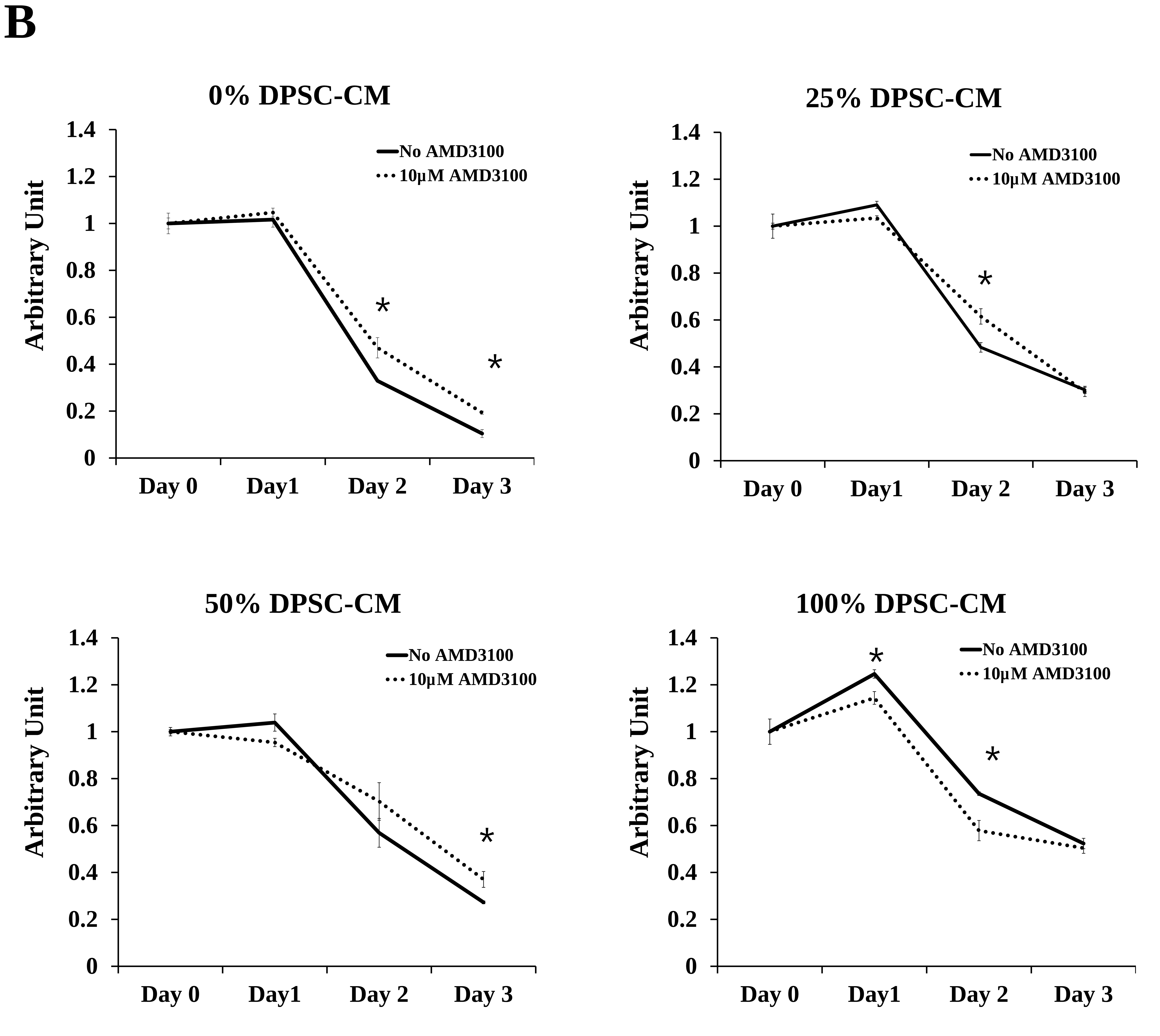

Supplementary Table 1. Antibodies for FACS analysis

Antibodies	Conjugation	Company	Dilution
CD14	FITC	BD	1:100
CD29	PE	BD	1:100
CD31	FITC	BD	1:100
CD34	APC	BD	1:100
CD44	APC	BD	1:100
CD45	FITC	BD	1:100
CD73	PE	BD	1:100
CD90	FITC	BD	1:100
CD117	PE	BD	1:100
HLA-I	FITC	BD	1:100
HLA-DR	APC	BD	1:100
Chondroitin Sulfate (NG2)	Alexa Fluor 488	BD	1:100
CD140b (PDGFRβ)	PE	BD	1:100
CD146	Alexa Fluor 647	BD	1:100

Supplementary Table 2. Primers for qPCR

Genes	Size (bp)	5' Oligonucleotide	3' Oligonucleotide	Tm (°C)
NG2	277	ACTGGCTAGGGGTGTCAATG	TCCTCAAGGTCCTGCTGAGT	60
α-SMA	256	CCGGGAGAAAATGACTCAAA	GAAGGAATAGCCACGCTCAG	60
PDGFRβ	286	GACAGGGAGGTGGATTCTGA	AGGTGTAGGTCCCCGAGTCT	60
CD146	101	ACTTCCACCTCCACCAGCTC	GTCTGCGCCTTCTTGCTC	60
PDGF-BB	105	CTGGCATGCAAGTGTGAGAC	AATGGTCACCCGAGTTTGG	63
VEGF	136	CACACAGGATGGCTTGAAGA	AGGGCAGAATCATCACGAAG	63
VEGFR1	110	TCCCTTCCTTCAGTCATGTGT	AAGAAGGAAACAGAATCTGCAA	60
VEGFR2	121	CGGCTCTTTCGCTTACTGTT	TCTCTCTGCCTACCTCACCTG	60
SDF-1α	108	TTGACCCGAAGCTAAAGTGG	TGGGCTCCTACTGTAAGGGTT	60
CXCR4	145	GGAGGGGATCAGTATATACA	GAAGATGATGGAGTAGATGG	60
GAPDH	208	GATGCTGGCGCTGAGTACG	GCTAAGCAGTTGGTGGTGC	60
